# Supplementary material for: Patterns of Intron Gain and Loss in Fungi
Source: PLoS Biol. 2004 Nov 30;2(12):e422. doi: 10.1371/journal.pbio.0020422 (PMC532390; doi:10.1371/journal.pbio.0020422)
Supplement: Table S1 — Also available at http://genes.mit.edu/NielsenEtAl/. (4.3 MB ZIP). [file pbio.0020422.st001.zip › NielsenEtAl/html/183.html]

AN5742.1.NCU05897.1.MG09193.1.FG01593.1


```
 CLUSTAL W (1.82) Multiple Sequence Alignments - Introns Inserted


Sequence 1: MG09193.1	477 aa
Sequence 2: FG01593.1	466 aa
Sequence 3: NCU05897.1	471 aa
Sequence 4: AN5742.1	459 aa
Alignment Length: 480 aa
Number Identitical Residues: 214 aa
Alignment Score (without introns) 11191


MG09193.1 	MFTREWWAKRR-LKVQDDKTTKAAELTLRQSIFPIFLVTILFFLW~GFSYGLLDTLNKHF
NCU05897.1	MFSREWWKRRS-LRVRDDKVTKAAELSLRESLLPLCLVTILFFLW~GFSYGLLDTLNKHF
FG01593.1 	MGVKQFFKDRP-LKVKDSKVTKAAELTLRESIWPIALVTVLFFLW~GFSYGLLDTLNKHF
AN5742.1  	MGFVRFLKTRSGLRVDNTKTTSAANLTLRQSLWPLMLVTILFFLW0--------------
          	*   .:   * .*:* : *.*.**:*:**:*: *: ***:*****               

MG09193.1 	QDVLDITRARSAGLQAAYFG2AYPLASLGHAAWILRHYSYKAVFIWGLFLYG1VGALLCI
NCU05897.1	QETLHITKARSAGLQAAYFG2AYPLASLGHAAYILRRFSYRAVFIWGLFLYG~LGALLAI
FG01593.1 	QKVLDIDRARSAGLQAAYFG2AYPLASLGHAAWILRHYSYKAVFIWGLSLYA~IGALIAI
AN5742.1  	-VTLNITRTRSSGLQAAYFG2AYPLASLGYANWLLRHYGYKLVFIFGLVLYG~IGALCMW
          	  .*.* ::**:******** ********:* ::**::.*: ***:** **. :***   

MG09193.1 	PAILGRSFAGFCMGIFIIGNGLGSLETAANPFITV~CGPPRYSEMRINLSQAFNGIGTVI
NCU05897.1	PCIKAKSFAGFCVCIFIIGNGLGSLETAANPYITV~CGPPKYSEIRINFAQAFNGIGTVV
FG01593.1 	PCIKAKSFAGFCMSIFIIGNGLGSLETAANPFITV1CGPPRYSEIRINISQAFNGVGTVI
AN5742.1  	PAGLNRSFGGFCAATFVIGSGLGSLETAANPYLAV1CGPPKYAEIRINLAQAFNGIGTVV
          	*.   :**.***   *:**.***********:::* ****:*:*:***::*****:***:

MG09193.1 	APVMGSYVFFRALDDKA-ALRNVQWVYLAIACFVFSLAVVFLLSDIPEITDAD~MARQAE
NCU05897.1	APVLGSYVFFG-FDDNL-ALQNVQWVYLAIAVFVYILAVVFFLIELPEITDAD~MQHQAA
FG01593.1 	APVLGSYVFFNNLDDEE-ALANVQWVYLSIAVFVLILACLFYISKIPEITDAD1MAFQAQ
AN5742.1  	APALASYVFFTDTEDSVDALKRVQWVYLAIGIFVFVLAAVFFMSTIPEVTDQD~MAFQVA
          	**.:.*****   :*. .** .******:*. **  ** :* :  :**:** * *  *. 

MG09193.1 	ETHAGEGDQPFRKQYRLFHAAFAQFCYTGAQV~AVASFFINYAVETLPGTDSAVGAQLFA
NCU05897.1	ETHAGDADQPFRKQYRLFHASFSQFCYTGAQI~AIAGYFINYVTETRKNTDSALGAQFLA
FG01593.1 	ETHSKADEKPFIKQYKLFHASFAQFCYTGAQV~AIASFFINYAVELRPNTSESVGAKLFA
AN5742.1  	TTHVDEQDKPFWKQYKLFHATFAQFTYTGAQV1AIASYFINYATETWPAVDNSTGSKYLA
          	 **    ::** ***:****:*:** *****: *:*.:****..*    ...: *:: :*

MG09193.1 	GGQAAFTVGRFSGVLFMHYFKPRLVFGGYLASVCVFLIPAIVLRGNAAIAMLFIVLFFES
NCU05897.1	GAQGTFAVGRFAGAAIMHFVRPRKVFLLFLTACIIFVAPTITQRENTGMSLLYVTLFFES
FG01593.1 	GAQAAFTVGRFFGTFLMKFIKPRKVFLVFLTMCIVFIGPPIVHHGNAGVACLYVVLFFES
AN5742.1  	GAQAAFTVGRFLGAFLMKYIRPRWVFLGYISGVVAFCAASTTQRNETGVAMLFLTLFFES
          	*.*.:*:**** *. :*::.:** **  :::    *  .. . : ::.:: *::.*****

MG09193.1 	ICFPTIVALGMRGLGRHSKRGSGLIVGGVLGGAVVPPLTGAVADIH-STPTAMVVPLAFM
NCU05897.1	ICFPTIMALGMRGLGRHTKRGSGFLVAGVFGGAVVPPLMGAVADMH-DTAMSMVVPLAFF
FG01593.1 	ICFPTIVALGMRGLGRHTKRGSGFIIAGVIGGACVPPLTGAVADMH-SMGISMVVPMVFF
AN5742.1  	VCFPTIVALGIRGLGRHYKRGSGFIVGGVSGGAAIPPLLAHVADLRNDTGFAFIVPTMFM
          	:*****:***:****** *****:::.** *** :*** . ***::..   :::**  *:

MG09193.1 	IAAMTYAVAVNFHPWYRDTADAFTAAEIGIKPRTDGEEALEKGVVHDHDEKRTEEKRVEA
NCU05897.1	AVAWTYALAVNFWPWYRDTCDAFTTAEIGVRDREDG--VVSKSVVDEEEAVGMEEQTVGE
FG01593.1 	VAALSYPIAVNFVPAYKNVVDVFYEADIGIGDRTLDEEKIQGGQIEEKGE-------LPA
AN5742.1  	VLAWTYAVAVNFVPAYRDTVDKVGSSDIGLTGSALDNGGKKDGKDVEALGVVEKGEVVHV
          	  * :*.:**** * *::. * .  ::**:     ..   . .   :      . . :  

MG09193.1 	AMANPL
NCU05897.1	KGL---
FG01593.1 	AR----
AN5742.1  	------
          	
```
